# Proliferation and Differentiation of Intestinal Caco-2 Cells Are Maintained in Culture with Human Platelet Lysate Instead of Fetal Calf Serum

**DOI:** 10.3390/cells10113038

**Published:** 2021-11-05

**Authors:** Dalanda Wanes, Hassan Y. Naim, Franziska Dengler

**Affiliations:** 1 Institute for Biochemistry, University of Veterinary Medicine Hannover, Foundation, D-30559 Hannover, Germany; dalanda.wanes@tiho-hannover.de (D.W.); hassan.naim@tiho-hannover.de (H.Y.N.); 2 Institute of Veterinary Physiology, Leipzig University, D-04103 Leipzig, Germany; 3 Institute of Physiology, Pathophysiology and Biophysics, University of Veterinary Medicine Vienna, A-1210 Vienna, Austria

**Keywords:** 3R, cell culture, differentiation, FCS, hPL, gene expression, morphology, protein expression, synthetic FCS

## Abstract

Cell lines are widely used as in vitro model systems and substitute for animal experiments. The frequently used Caco-2 cell line is considered to reflect characteristics of differentiated intestinal epithelium. However, the need to culture the cells with fetal calf serum (FCS) induces a high variability, risk of contamination and is ethically disputed. We tested the culture of Caco-2 cells with human platelet lysate (PL) instead of FCS. We compared cell viability and differentiation by measuring ATP levels, gene and protein expression of specific markers in total cell extracts, brush border membrane vesicles (BBM) and lipid rafts (LR). Cell viability was slightly enhanced in cells grown with PL compared to FCS. The cells differentiated to an intestinal phenotype like the cells cultured in FCS, as indicated by the similar gene expression levels of hexose and protein transport proteins and the structural protein *VILLIN*. BBM showed a comparable distribution of the intestinal hydrolases, indicating a maintained cell membrane polarity. The distribution of the marker protein FLOTILLIN-2 in LR was also similar. We conclude that PL is an exquisite and suitable replacement for FCS in the culture of Caco-2 cells that can eliminate many disadvantages incurred due to the use of FCS.

## 1. Introduction

Cell culture experiments are an important tool for basic science, pharmacology, toxicology, and even clinical applications [1]. Besides the high reproducibility and easy handling, the use of cell lines is also ethically superior to animal experiments according to the 3R (reduce, refine, replace) principles. However, to ensure growth and proliferation of most cell types, the media for their cultivation must be supplemented with different factors. This requirement is usually fulfilled by the addition of fetal calf serum (FCS). FCS consists of an undefined cocktail of growth factors, vitamins, hormones and, in contrast to adult serum, low immunoglobulin levels, which may otherwise inhibit cell growth [2]. FCS is harvested from bovine fetuses at the abattoir and as the level of fetal awareness is unknown, it is also not clear whether or not the unborn calves experience distress or even suffer from the procedure [3,4]. It is assumed that the worldwide demand for FCS in research applications is approximately 500,000 L per year, which equals 1,000,000 fetuses [4,5].

Besides the ethical precariousness, the use of this animal-derived product infers several other disadvantages. The variation in the composition and quality of different batches of FCS hampers the reproducibility and standardization of cell culture experiments. Additionally, FCS might even contain endotoxins, mycoplasma, or other infectious agents, leading to a contamination or death of the cells. Furthermore, the culture of cells for therapeutic use in patients requires an allogenic background, i.e., without supplements of animal origin. All of this renders a replacement of FCS desirable. 

Many attempts have been made to culture cells under serum-free conditions, but only few of them have been successful. Likewise, several efforts have been made to generate synthetic supplements with a composition comparable to that of FCS. Due to the specific needs of various cell types, the establishment of well-defined media has not made significant progress yet [6]. Obviously, growth factors and hormones are not the only components in FCS that are essential for cell growth but may be found in other blood products as well. Several variants have been tested, ranging from neonatal calf serum to adult bovine or porcine serum, which still infer the death of donor animals. A promising serum-derived product are blood platelet lysates (PL). This supplement can be easily produced from overaged transfusion products by freeze–thaw cycles and is even superior to FCS in terms of biosafety and production cost [7]. However, for many years, human PL has been mainly used in the culture of mesenchymal stem cells [8,9] and is still barely known among researchers using conventional cell cultures. Recently, the suitability of PL for the culture of several immortal cell lines has been investigated. It was demonstrated that cell lines with myeloma, leukemia, fibroblast and also epithelial background can also be grown with PL instead of FCS as medium supplement [10,11,12,13,14]. 

Caco-2 cells are derived from colon carcinoma and are widely used as a model for intestinal epithelial cells [15,16]. Due to their close resemblance to enterocytes, they have been increasingly used as an in vitro model for high-throughput screening assays in the last decades for testing intestinal drug absorption and mechanisms for drug transport [16,17,18]. They have been shown to express the full set of transport proteins and intestinal hydrolases characteristic for the small intestinal epithelium [17], although not exactly at the same expression levels [18].

A search in PubMed (National Center for Biotechnology Information (NCBI), Bethesda, MD, USA) for Caco-2 resulted in over 21,000 hits with an exponential increase from 1979 to 2020; in 2020 alone over 1600 studies using this cell line were published and most of these studies used FCS for their cultivation. Replacing FCS in the experiments with Caco-2 cells alone would be a significant step towards a better implementation of the 3R. To our knowledge, PL has never been used in the culture of Caco-2 cells instead of FCS. Additionally, most studies that tested the culture of immortal cell lines with PL focused on cell proliferation and morphology but there is limited data on the differentiation characteristics of cells in PL as compared to FCS supplemented media. 

In this study, we compared several supplements, both synthetic and natural, for their eligibility in the culture of Caco-2 cells with particular emphasis on cell growth, viability, and differentiation. Strikingly, only PL maintained the biological features of Caco-2 cells in a manner similar to the cells cultured in FCS. Our results strongly recommend the use of PL instead of FCS in future studies with Caco-2 cells.

## 2. Materials and Methods

### 2.1. Cell Culture

Human colon carcinoma Caco-2 cells from the German Collection of Microorganisms and Cell Cultures (DSMZ, Braunschweig, Germany) were used in passages 20–35. The cells were preserved in liquid nitrogen. For the experiments, one portion was thawed quickly, seeded in a T75 flask (Greiner Bio One, Frickenhausen, Germany) with conventional medium (see below) and grown to confluency at 37 °C in a humidified atmosphere (5% CO_2_ + 95% air). Then, the cells were split with trypsin (Sigma–Aldrich, Darmstadt, Germany) and reseeded in T25 flasks or 96-well plates according to the respective downstream analysis and cultured with the different media compositions for comparison. 

The culture medium was composed of high-glucose DMEM, 3 mM L-glutamine, 100 U/mL penicillin/streptomycin and was supplemented with 10% FCS (Sigma-Aldrich, Darmstadt, Germany) for the conventional medium or 10% of one of the following synthetic or PL-supplements:

- Panexin Basic Serum Replacement with Defined Components (P04-96090, PAN Biotech, Aidenbach, Germany); “basic”
- Panexin NTA Serum Substitute with Defined Components for Adherent Cells (P04-95750, PAN Biotech, Aidenbach, Germany); “NTA”
- PL solution research grade (PLS-100.01PL BioScience GmbH, Aachen, Germany); “hPL”
- PL solution-FD (fibrinogen derived) research grade (PLS-FD-500.01, PL BioScience GmbH, Aachen, Germany); “FD”
- PL solution-XF (fibrinogen derived, xenogen-free) research grade (PLS-XF-500.01, PL BioScience GmbH, Aachen, Germany); “XF”

The medium containing hPL was additionally supplemented with heparin (PL SUPPLEMENT, PL-SUP-500, PL BioScience GmbH, Aachen, Germany) according to the manufacturer’s instructions.

Phase contrast microscopy was used to observe cell growth and morphology. 

### 2.2. Viability Assay

Cell viability was assessed using the Cell Titer Glo^®^ Luminescent Cell Viability Assay (Promega GmbH, Walldorf, Germany) according to the manufacturer’s instructions. Shortly, cells were grown in opaque 96 well plates with the different supplements for six days. Then, the medium was removed and after washing with phosphate-buffered saline (PBS) (pH 7.4) 100 µL CellTiter-Glo^®^ Reagent were added to each well, mixed by shaking the plate for 2 min and incubated at room temperature for 10 min. Subsequently, the luminescence of each well was measured with a GloMax^®^ 96 Microplate Luminometer (Promega GmbH, Walldorf, Germany).

### 2.3. Gene Expression Analysis: Two-Step RT-qPCR

The cells grown in T25 flasks were washed with 4 °C PBS once and harvested mechanically approximately 5 days after reaching confluency. After spinning down at 800× *g* for 5 min, the cell pellet was resuspended, and cells were counted using a trypan blue live/death staining. Then, two aliquots of 1 × 10^6^ cells were washed with PBS once more, spun down again and the pellets were snap frozen and stored at −80 °C.

For the extraction of total RNA, the pellets were thawed in lysis buffer and further processed with the ReliaPrep™ RNA Miniprep System (Promega GmbH, Mannheim, Germany) following the manufacturer’s protocol. The RNA concentration and quality were determined using a spectrophotometer (BioPhotometer, Eppendorf, Wesseling-Berzdorf, Germany). 1 µg of high-quality RNA was used for cDNA synthesis using the GoScript^TM^ Reverse Transcriptase Kit (Promega, Mannheim, Germany) according to the manufacturer’s instructions using a MJ Research PTC-200 Peltier Thermal Cycler (Bio-Rad, Feldkirchen, Germany). 

For qPCR, the resulting cDNA was diluted 1:20 and 2µL were used in a 20 µL reaction volume containing 10 µL of a ready-to-use premix of SYBR Green I dye, dNTPs, stabilizers, and enhancers (GoTaq^®^, Promega GmbH, Mannheim, Germany), 112 nM primer mix and DNase-free water. These mixtures were pipetted in strip tubes (0.1 mL Strips, LTF Labortechnik, Wasserburg, Germany) and processed in a Corbett Rotor-Gene 6000 (Qiagen, Hilden, Germany) at individually optimal protocols (Table 1). A no template control (NTC) with DNase-free water instead of cDNA was applied for each run. qPCR reactions for each sample and gene were run in duplicate to minimize dispensation artefacts. The deviation of C_t_ of the technical replicates was <0.3. If it was higher, data were discarded, and the run was repeated. The PCR cycles were run using automatic fluorescence emission following each PCR cycle, and the amplification specificity was checked after each run by melting curve analysis. The primer sequences and conditions for qPCR are shown in Table 1; the denaturation temperature was always 95 °C and the extension was performed at 60 °C. 

The primers were designed with the Primer BLAST tool from the NCBI (Bethesda, MD, USA) according to known sequences from the Basic Local Alignment Search Tool (BLAST) in the gene bank database of the NCBI and synthesized by Eurofins MWG (Ebersberg, Germany). The amplicons were sequenced again, and the product sequences were verified by BLAST. 

The quantification cycle and amplification efficiency of each amplification curve were determined using the Rotor Gene 6000 Series Software 1.7 (Corbett/Qiagen, Hilden, Germany). For analysis of the data, the ΔΔC_t_ method was used to compare the mRNA expression. The C_t_ values set by the software were applied after checking them optically. 

Normalization of the samples was achieved using the same amounts of RNA for processing and by normalizing the data for the target genes with the aid of the reference genes hypoxanthine guanine phosphoribosyltransferase 1 (*HPRT1*) and peptidylprolyl-isomerase A (*PPIA*). Therefore, the geometric mean of both reference genes’ C_t_ values was calculated and used for normalization. The reference genes have been proven to be stable under the experimental conditions applied in our study. Their stability was tested using the programs BestKeeper© (Version 1 by M.W. Pfaffl, Institute of Physiology, Center of Life and Food Sciences, TUM-Weihenstephan, Munich, Germany, 2004) and geNorm [19].

### 2.4. Protein Expression Analysis

#### 2.4.1. Extraction of Total Protein

For total protein extraction, the cells were treated as described for the mRNA extraction and approximately 2 × 10^6^ cells were stored at −80 °C until the cell pellet was resuspended in 500 µL of a lysis buffer consisting of 10 mM EDTA, 4 mM EGTA, 50 mM Tris buffer, 100 mM β-glycerin-phosphate-disodium pentahydrate, 0.1% Triton X 100, 15 mM sodium orthovanadate, 100 mM sodium pyrophosphate tetrabasic decahydrate, and 2.5 mM NaF (pH 7.4) (all chemicals: Sigma–Aldrich, Darmstadt, Germany) with a protease and phosphatase inhibitor (100X Halt^TM^ protease and phosphatase inhibitor cocktail, Thermo Fisher Scientific, Dreieich, Germany). The protein concentration was measured with a Tecan Spectra Rainbow Microplate Reader (Tecan Deutschland, Crailsheim, Germany) using the bicinchoninic acid method and bovine serum albumin as standard.

#### 2.4.2. Preparation of Brush Border Membranes (BBM)

The isolation of BBM was carried out with the modified divalent cation precipitation method, according to Schmitz et al. [20] and as modified by Sterchi and Woodley [21]. Shortly, the cells were homogenized in homogenization buffer (12 mM Tris-HCl, pH 7.0, 300 mM mannitol) and protease inhibitor mix (1.48 μM antipain, 1.46 μM pepstatin A, 10.51 μM leupeptin, 0.768 μM aprotinin, 50 μg/μL soybean trypsin inhibitor, and 1 mM phenylmethylsulfonyl fluoride; all were obtained from Sigma–Aldrich, Darmstadt, Germany) using a Potter–Elvehjem homogenizer followed by ultrasonic pulses. After centrifugation at 5000× *g* for 15 min at 4 °C, the obtained homogenates (H) were precipitated using 100 mM CaCl_2_. After 30 min rotation at 4 °C, a centrifugation of 20 min at 5000× *g* was applied to yield the intracellular and basolateral membranes (P1) and the supernatant (S). A final centrifugation at 25,000× *g* for 30 min at 4 °C was performed in order to yield the BBM (P2). The collected pellets, P1 and P2, were dissolved in the homogenization buffer and subsequently analyzed by Western blot.

#### 2.4.3. Preparation of Lipid Rafts (LR)

The isolation of detergent resistant membranes, i.e., LR, was conducted on a discontinuous sucrose density gradient [22]. To do so, the Caco-2 cells were homogenized at 4 °C for 2 h with 1% (*w/v*) Triton X-100 prepared in phosphate-buffered saline (pH 7.4) and a protease inhibitor mix (see above). Cellular debris were removed after centrifugation at 5000× *g* for 20 min at 4 °C. Thereafter, the cellular lysates were loaded on a sucrose gradient consisting of 1 mL 80% sucrose (*w/v*), 1 mL lysates in 40% sucrose (*w/v*), 7 mL 30% sucrose (*w/v*) and 1 mL 5% sucrose (*w/v*). The gradient was subjected to an ultracentrifugation at 100,000× *g* for 18 h at 4 °C using an SW40 rotor (Beckman Coulter, Mississauga, ON, Canada). Finally, 10 fractions (1 mL each) were harvested from the top to the bottom of the gradient where the 3 top fractions correspond to the LR and the 3 last bottom fractions are considered the non-lipid raft fractions (non-LR). The 10 fractions were subsequently analyzed for the distribution of sucrase–isomaltase (SI) in addition to a LR marker, FLOTILLIN-2.

#### 2.4.4. Western Blot Analysis

For Western blot analysis, the protein samples were separated by sodium dodecyl sulphate-polyacrylamide gel electrophoresis (SDS-PAGE) using 10 µg protein/well. Subsequently, the samples were transferred onto a PVDF (BBM and LR) or a nitrocellulose (total protein) membrane (Carl Roth, Karlsruhe, Germany) using the Mini-Protean© system (Bio-Rad laboratories, Feldkirchen, Germany). The membrane was preincubated in 5% skimmed milk (BBM and LR) or bovine serum albumin (total protein) in TRIS-buffered saline containing 0.2% Tween-20 (TBST) at 4 °C overnight. The next day, it was incubated with the primary antibodies (see Table 2) at room temperature with gentle shaking for 2 h. After washing with TBST five times, the membranes were incubated with an HRP-coupled secondary antibody (see Table 2) at room temperature with gentle shaking for 1 h. Subsequently, the membranes were rinsed again with TBST five times and once with TBS, then the signal was detected by enhanced chemiluminescence using a G:BOX Chemi XT4 and analyzed with the GeneTools© software (version 4.3.9.0, for total protein, both Syngene, Cambridge, UK) or with a ChemiDoc XRS System (for BBM and LR, Bio-Rad, Hercules, CA, USA). β-ACTIN was used as loading control for normalization of each quantitative blot (total protein). 

### 2.5. Statistics

The results are described as box plots representing the median ±10th, 25th, 75th and 90th percentile. The significance is expressed as the probability of error (*p*). Each independent experiment with different stocks and passages of the cells is considered as biological replicate (“N”), while technical replicates are considered as “*n*”. The technical replicates were pooled for each N for statistical analysis, except for the cell viability assay. The differences between the mean values of the cells cultured with a supplement and FCS were assessed using a paired *t*-test or a one-way repeated measurements (RM) ANOVA with subsequent Holm–Sidak test for comparison of more than two groups (Sigma Plot 13.0, Systat Software, San Jose, CA, USA). The differences were assumed to be statistically significant if *p* < 0.05.

## 3. Results

### 3.1. Morphology

**Figure 1:**
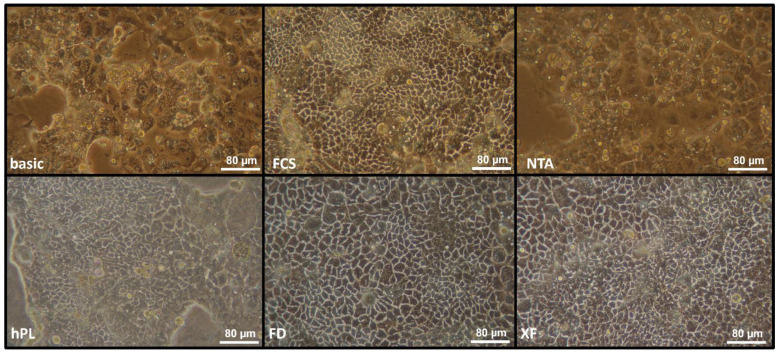
Representative pictures from phase contrast microscopy of Caco-2 cells after 7 days in culture with different supplements (as indicated in each picture, abbreviations see Section 2.1).

The morphology of the cells was assessed during growth with different media supplements using phase contrast microscopy. Figure 1 shows representative images 7 days after seeding the cells. Cells cultured with the synthetic supplements (basic and NTA) appeared less vital and proliferated more slowly compared with those in FCS. Additionally, they never became confluent and showed signs of senescence. Thus, we did not conduct further analyses with these conditions.

In contrast, cells cultured with PL preparations displayed a similar morphology like cells grown in FCS. Especially with FD and XF, cells seemed to proliferate even faster than cells grown with FCS, but the cell number counts did not differ significantly. 

### 3.2. Cell Viability

**Figure 2:**
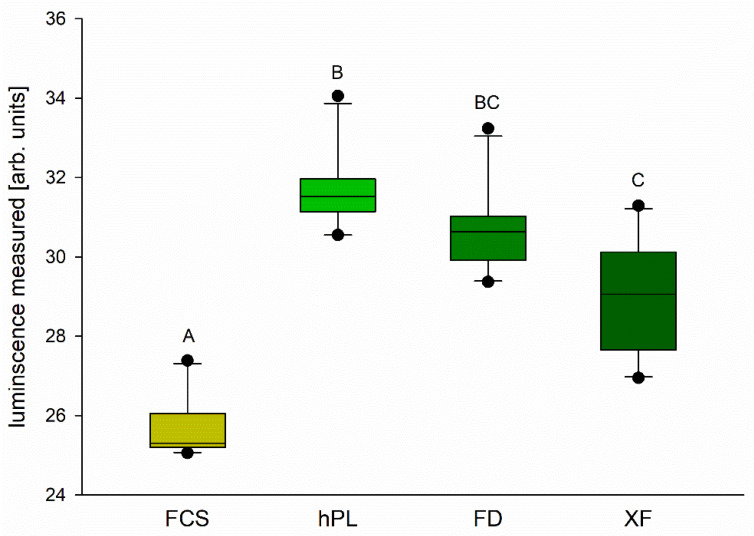
Cell viability was assessed using a Cell Titer Glo^®^ Luminescent Cell Viability Assay. Boxes represent medians ±10th, 25th, 75th and 90th percentile. Outliers are represented by dots; *n* = 10, one-way RM ANOVA + Holm–Sidak test, *p* < 0.01. Different letters (A, B, C) indicate significant differences between groups.

Due to the morphology and proliferation of the cells in synthetic media, the cell viability was tested only in cells grown with FCS or different PL. Comparing the ATP content of the confluent cells, we found significantly higher values in all PL supplements compared to FCS (Figure 2). Furthermore, hPL seemed to be superior to FD or XF (Figure 2). 

### 3.3. Gene Expression

**Figure 3:**
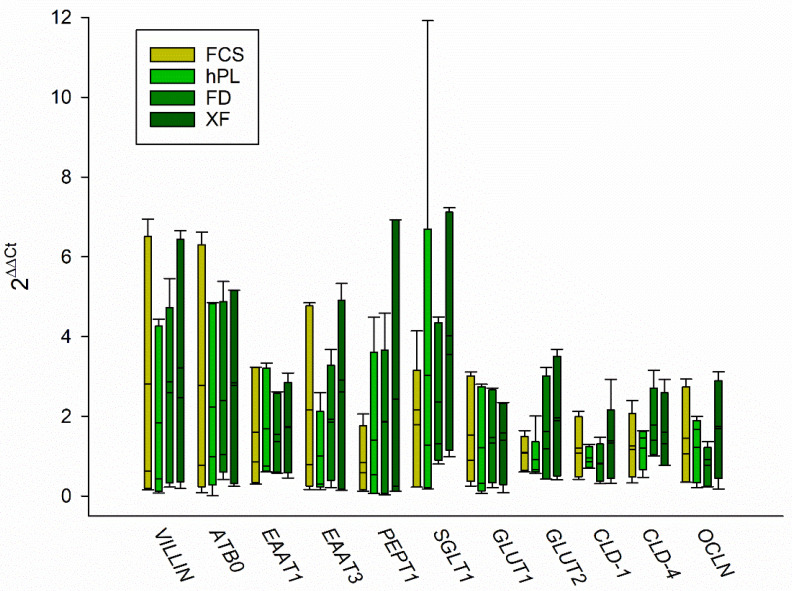
Gene expression was measured using RT-qPCR to assess differentiation of the cells with the different supplements compared to cells grown with 10% FCS in the culture medium. Boxes represent medians ±10th, 25th, 75th and 90th percentile; N = 5; One-Way RM ANOVA. *ATB0*: amino acid transporter B0, *EAAT*: excitatory amino acid transporter, *PEPT1*: peptide transporter 1, *SGLT1*: Na+-coupled glucose transporter 1, *GLUT*: glucose transporter, *CLD*: claudin, *OCLN*: occludin.

In order to evaluate the grade of differentiation of the cells with different media supplements, we measured the gene expression of markers for differentiated enterocytes using RT-qPCR. As a major function of a differentiated enterocyte is the vectorial transport of nutrients, we assessed the expression of the amino acid transporter B0 (*ATB0)*, excitatory amino acid transporter (*EAAT*) 1 and 3 and peptide transporter (*PEPT*) 1 as well as the glucose transport proteins sodium linked glucose transporter (*SGLT*) 1 and glucose transporter (*GLUT*) 1 and 2. Similarly important is the ability to form a barrier, which is why we also assessed the expression of the tight junction proteins claudin (*CLD*) 1 and 4 as well as occluding (*OCLN*). Additionally, the brush border membrane protein *VILLIN* was chosen. In all PL supplements, all genes investigated were expressed in a similar range and there was no significant difference to those cultured with FCS (Figure 3).

### 3.4. Protein Expression

**Figure 4:**
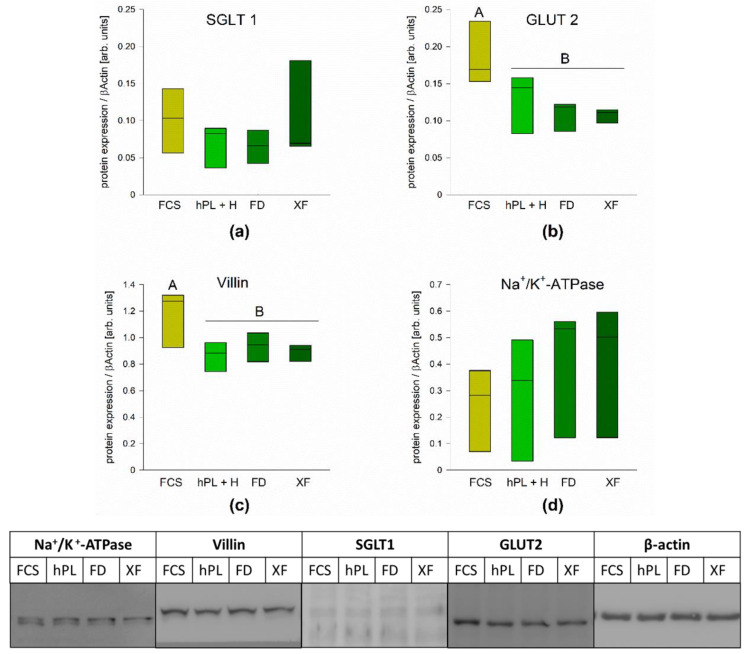
Protein expression of SGLT1 (**a**), GLUT2 (**b**), v (**c**) and Na^+^/K^+^-ATPase (**d**) was assessed by Western blot analysis in order to evaluate differentiation of the cells after growth with the different supplements. Boxes represent medians ±10th, 25th, 75th and 90th percentile; N = 3, one-way RM ANOVA + Holm–Sidak test, *p* < 0.05. Different letters (A, B) indicate significant differences between groups. Representative blots are shown below. GLUT: glucose transporter, SGLT1: Na+-coupled glucose transporter 1.

As the mRNA expression does not always allow for direct conclusions on the abundance of the functional protein, we also measured the protein expression of some markers in the FCS and PL supplemented cells via Western blot. Our results revealed a significantly reduced expression of VILLIN and GLUT2 in all PL supplemented cells compared to FCS (Figure 4b,c), whereas the protein levels of SGLT1 and Na^+^/K^+^-ATPase were similar in all groups (Figure 4a,d).

**Figure 5:**
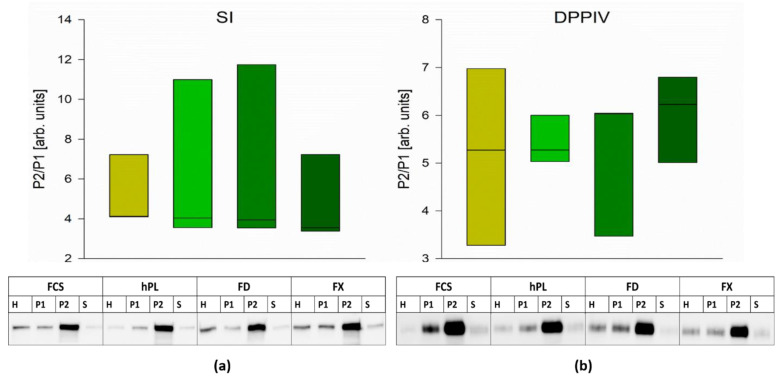
The expression of sucrase–isomaltase (SI) (**a**) and dipeptidyl-peptidase (DPPIV) (**b**) in BBM was assessed by Western blot analysis. To evaluate the grade of protein trafficking to the apical membrane, the ratio between P2 and P1 was calculated and is shown in the graph. Boxes represent medians ±10th, 25th, 75th and 90th percentile; N = 3; one-way repeated measurements ANOVA. Representative blots are shown below. H: cellular homogenate; P1: intracellular and basolateral membrane; P2: BBM; S: soluble and vesicular (endosomal) proteins.

To further examine the correct sorting or trafficking of brush border membrane proteins, we prepared BBM and measured the protein expression of dipeptidyl-peptidase IV (DPPIV) and sucrase–isomaltase (SI). As shown in Figure 5, the sorting of SI (Figure 5a) or DPPIV (Figure 5b) within the groups cultured with the different media supplements was similar to the group cultured with FCS.

**Figure 6:**
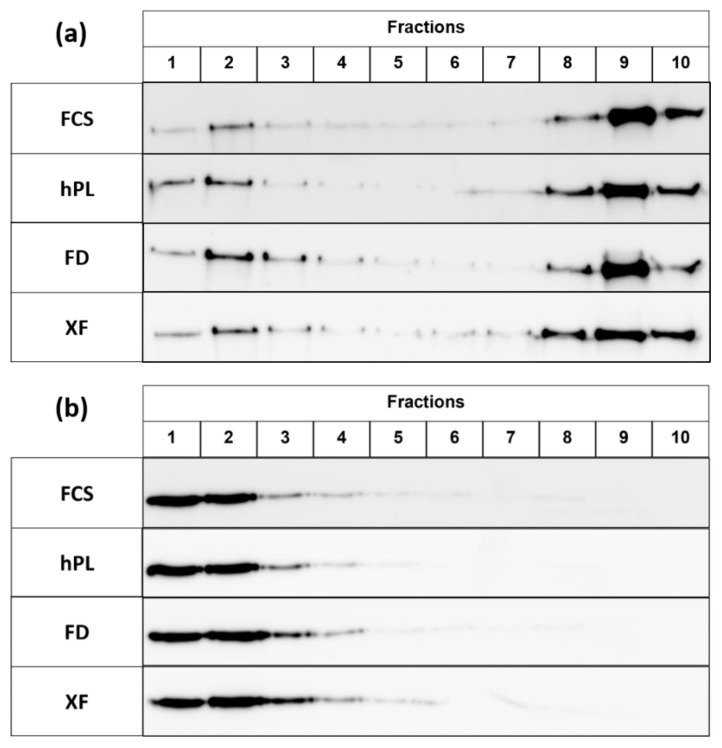
The expression of sucrase–isomaltase (SI) (**a**) and FLOTILLIN-2 (**b**) in lipid rafts (LR) was assessed by Western Blot analysis and compared qualitatively. The figure shows a representative blot of N = 3. There were no differences regarding the localization of the proteins between the different supplements.

The detection of SI and FLOTILLIN-2 in LR further confirmed that the membrane trafficking is similar in PL supplemented and FCS supplemented cells (Figure 6a,b).

## 4. Discussion

Many areas of research depend on the use of immortal cell lines representing the respective tissue as closely as possible while being easy to handle (also in large scale) and offering a standardized and reproducible matrix for manifold applications ranging from drug toxicity testing to complex genetic manipulations. To ensure the translation of in vitro results in cell lines to their behavior in organs in vivo, their characteristics must resemble those of the tissue that they are supposed to represent throughout the culture period. It is a well-accepted fact that the supplementation of cell culture media with growth factors is necessary to support the proliferation of the cells and that these supplements may influence cellular growth and differentiation. Despite many attempts to substitute this non-standardized and ethically disputed product with synthetic supplements or serum-free media, FCS is still used in most cell or tissue culture experiments [25]. The failure of Caco-2 cells to proliferate and differentiate when grown with synthetic supplements, as shown in this study, does not favor the utilization of these supplements to address questions on the cellular and functional levels in these cells relevant to intestinal physiology. Recently, PL attracted interests as a supplement of FCS, since it has been shown to support the proliferation of mesenchymal stem cells even more efficiently than FCS [9,26]. Since then, culture of several cell lines with PL instead of FCS has been tested, but to our knowledge, our study is the first of its kind in which the suitability for the frequently used Caco-2 cell line was examined. While most studies have revealed virtually similar proliferation and viability profiles of all the cell strains tested, the effects on the cell differentiation have rarely been investigated. 

We tested two synthetic supplements and three different PL preparations for their performance in the culture of Caco-2 cells. While the synthetic supplements did not maintain cell proliferation and differentiation, PL supports cell growth, propagation, and differentiation similar to or even outperforms FCS. Cells grown in media containing synthetic supplements did not proliferate or acquire confluence in a fashion similar to their counterparts in FCS as assessed by phase contrast microscopy, rendering further detailed analyses unnecessary. Hitherto, we focused our studies on the effects of different PL preparations.

The preparations used differed in their grade of purification. The simplest one, hPL, contains fibrinogen so that heparin must be added to the medium to prevent clotting. Heparin might impair cellular proliferation at certain concentrations [27]. This is amended in FD, i.e., fibrinogen-depleted PL, and XF, which is also a xenogen-free preparation and may be used for the culture of cells for therapeutic applications. Overall, we found no differences in the performance of the different preparations. 

In fact, the cells cultured in the presence of hPL, FD or XF grow at least as well as those cultured with FCS if not better. This is in accordance with reports of hematopoietic cells having shorter generation intervals when cultured with PL [14]. Furthermore, the viability was even higher in cells supplemented with hPL, FD and XF as compared to FCS. The higher ATP levels that are indicative of increased cell viability could be due to a slightly higher cell number due to a higher proliferation rate in the tested groups. Nevertheless, routine cell number counts that did not differ significantly between the groups exclude this possibility. A similar increase in cell proliferation and viability has been reported for human corneal endothelial cells cultured with human PL as compared to FCS [28].

The proliferation rate of a cell line might well be increased at the expense of cell differentiation. This notion is supported by the lower concentrations of GLUT2 and VILLIN, which we found on the protein level in comparison to cells cultured with FCS, while the expression of SGLT1 and Na^+^/K^+^-ATPase did not differ significantly. A recent study demonstrated that the activity of Na^+^/K^+^-ATPase is increased during the maturation of enterocytes [29], thus the increasing trend we observed for this protein in the Caco-2 cells cultured with PL might also be indicative of a higher differentiation level. Overall, the differences are minor; they could be due to the heterogeneity in Caco-2 cells [30] and may have no biological relevance. Since the differentiation levels of Caco-2 cells vary largely between passages and clones [16], it is important to emphasize that we compared cells subcultured from the same batch within identical time frames and culture conditions except for the media supplements for each biological replicate.

The mRNA expression levels in the cells grown with PL were comparable to those cultured with FCS, while slight variations in the protein expression levels could be associated with differences in the protein turnover due to increased cell viability and proliferation. An increasing expression and functionality of nutrient transporters, such as SGLT1, GLUT2, PEPT1 and EAATs as well as intestinal hydrolases, such as SI and DPPIV, was observed in Caco-2 cells, indicating an increased differentiation [16,24,31,32]. Thus, we conclude that the grade of differentiation was similar in cells cultured with PL compared to FCS supplemented cells. Furthermore, we investigated the polarity of the cells grown with PL and FCS in BBM and LR. The level of enrichment of intestinal hydrolases in the BBM of Caco-2 cells is concomitant with the level of differentiation and polarity in these cells [24]. We measured the expression of the marker proteins SI and DPPIV, which were distributed similarly in the subcellular fractions in PL and FCS supplemented cells, suggesting similar polarization and differentiation of the Caco-2 cells when cultured with PL or FCS. Moreover, SI associates with cholesterol- and sphingolipids-enriched microdomains or LR before it is sorted to the BBM [33,34]. Here again, an essentially similar distribution pattern of SI and flotillin-2 in LR was revealed, indicating that the sorting event is maintained under these culturing conditions. 

Previous attempts to culture Caco-2 cells under serum-free conditions revealed variations in the differentiation and functionality of the cells, such as a loss of brush border enzyme activities or a higher paracellular permeability, indicating a loss of epithelial tightness and polarity [15,30,35]. Caco-2 cells cultured in serum-free medium were also shown to express significantly lower levels of efflux transporters and tight junction proteins, and transepithelial resistance measurements confirmed a reduced epithelial barrier function [36]. At least on an mRNA level, we did not observe any differences in the expression of tight junction proteins in the cells cultured with PL compared to FCS. 

While the combination of human cells and human PL works naturally, several cells of animal origin do not thrive as well with human PL instead of FCS. Porcine PL has been shown to support the growth of CHO, Vero and hybridoma cells [37], indicating that xenogenic PL might work for some cell lines. More recently, the production of equine PL and its use in the successful culture of equine mesenchymal stem cells has been reported [38]. This opens completely new possibilities for allogenic supplementation of culture media for cells of different origin and thus not only an improvement from an ethical point of view but also improved culture conditions for individual cell lines. Even a clinical application of PL, e.g., in eye drops to enhance repair of corneal epithelial defects, may be an option [39].

While the use of PL eliminates the ethical issue, the poor standardization of the supplement remains a problem that cannot be solved completely using PL instead of FCS. With PL being a natural product like FCS, the variability between batches will remain an issue. It is crucial to further investigate and establish standards for PL production, such as determining the minimum pool sizes to eliminate donor-dependent effects [40]. By applying high standards throughout the collection and preparation process, as well as pooling of many batches, variation and contamination can be significantly decreased in comparison with FCS. It would also be desirable that the manufacturers disclose the information regarding the contents of each preparation to researchers to enhance reproducibility and foresee potential interactions. 

## 5. Conclusions

In summary, we could show that PL is well suited to replace FCS in the culture of Caco-2 cells without impairment of cell proliferation or differentiation. By contrast, synthetic supplements did not support cell growth sufficiently. While there were no differences between the performance of hPL, FD and XF, heparin might still inhibit cell growth under certain circumstances and thus, FD might be more reliable to produce consistent results. XF is intended to be used for clinical applications and its high grade of purification is not necessary for basic research. By using these supplements instead of FCS, cell culture models could finally become a real alternative to animal experiments.

## Figures and Tables

**Table 1:** Primers used for RT-qPCR.

Gene Name	Gene Bank Accession Number	Primer Sequence (5′–3′)	Annealing Temperature [°C]	Amplicon Length [bp]
*HPRT1*	NM_000194.3	F: ATGGACAGGACTGAACGTCTT R: TGTAATCCAGCAGGTCAGCA	57	118
*PPIA*	NM_021130.5	F: GCCGAGGAAAACCGTGTACT R: CTGCAAACAGCTCAAAGGAGAC	59	106
*SGLT1*	NM_000343.4	F: AAGACCACCGCGGTCAC R: AAACATAGCCCACAGTCCGA	57	119
*GLUT1*	NM_006516.4	F: GAACTCTTCAGCCAGGGTCC	60	113
		R: ACCACACAGTTGCTCCACAT		
*GLUT2*	NM_000340.2	F: CAATGCACCTCAACAGGTAATAA R: AGATTGTGGGCAGTTCATCTGT	57	119
*PEPT1*	NG_017032.1	F: GGTCCCACTGCTGCTTTTTG	60	117
		R: GCAGAGCAAGGGGTTGTACT		
*EAAT1*	NM_001166696.3	F: CCCCTTACAAAATCAGAAAAGTTGT	60	101
		R: GGAGGGTCTCTTCTTTGCACT		
*EAAT3*	NM_004170.6	F: GGTGGTGCTAGGCATTACCA	60	118
		R: TGATGAGTTTCAGCATCCGCA		
*ATB0*	NM_005628.3	F: GTGTCCTCACTCTGGCCATC	60	98
		R: TACAGGACCGGTCGACTAGC		
*VILLIN*	NM_007127.3	F: ACCGAGGGGATGTTTTCCTC R: GTCATGCCCCTGAGTCTCTC	60	93
*CLD 1*	NM_021101.5	F: CCAGTCAATGCCAGGTACGA	60	93
		R: ACAGCAAAGTAGGGCACCTC		
*CLD 4*	NM_001305.5	F: TCTCCTCTGTTCCGGGTAGG	60	90
		R: CAAGGCCTCAGCCATACTCC		
*OCLN*	NM_002538.4	F: GGTCTAGGACGCAGCAGATTG	60	112
		R: TGGACTTTCAAGAGGCCTGG		

F: forward primer; R: reverse primer; bp: base pairs; *HPRT1*: hypoxanthine guanine phosphoribosyltransferase 1, *PPIA*: peptidylprol-yl-isomerase A, *SGLT1*: Na^+^-coupled glucose transporter 1, *GLUT*: glucose transporter, *PEPT1*: peptide transporter 1, *EAAT*: excitatory amino acid transporter, *ATB0*: amino acid transporter B0, *CLD*: claudin, *OCLN*: occludin.

**Table 2:** Antibodies used for Western Blot.

Target	Primary Antibody	Manufacturer/Catalog Number	Dilution	Secondary Antibody	Manufacturer/ Catalog Number	Dilution
β-ACTIN	Mouse-anti-β-Actin	Santa Cruz Biotechnology/sc-47778	1:1000	Goat-anti-mouse HRP	Invitrogen/A16072	1:5000
DPPIV	Mouse-anti-Dipeptidyl peptidase (HBB 3/775/42)	Provided by Dres. E.E. Sterchi and H.-P. Hauri. University of Bern and University of Basel, Switzerland [23,24]	1:5000	Goat-anti-mouse HRP	Invitrogen/A16072	1:5000
GLUT2	Rabbit-anti-GLLUT2	Thermo Fisher Scientific/PA5-77459	1:200	Donkey-anti-rabbit HRP	Santa Cruz Biotechnology/sc-2077	1:10,000
Na^+^/K^+^-ATPase	Mouse-anti-Na^+^/K^+^-ATPase	Enzo Life Sciences AG 804-082	1:100	Goat-anti-mouse HRP	Invitrogen/A16072	1:5000
SGLT1	Rabbit-anti-SGLT1	Antibodies online/ABIN364451	1:1000	Donkey-anti-rabbit HRP	Santa Cruz Biotechnology/sc-2077	1:10,000
SI	Mouse-anti-sucrase–isomaltase (HBB3/705/60)	Provided by Drs. E.E. Sterchi and H.-P. Hauri. University of Bern and University of Basel, Switzerland [23,24]	1:5000	Goat-anti-mouse HRP	Invitrogen/A16072	1:5000
VILLIN	Rabbit-anti-Villin	Thermo Fisher Scientific/PA5-78222	1:500	Donkey-anti-rabbit HRP	Santa Cruz Biotechnology/sc-2077	1:10,000
FLOTILLIN-2	Mouse-anti-flotillin-2 B-6	Santa Cruz Biotechnology/sc-28320	1:5000	Goat-anti-mouse HRP	Santa Cruz Biotechnology, Inc., Texas, US	1:5000

DPPIV: dipeptidyl-peptidase IV, GLUT2: glucose transporter 2, SGLT1: Na^+^-coupled glucose transporter 1, SI: sucrase-isomaltase.

## Data Availability

The data presented in this study are available on request from the corresponding author.

## References

[1] van der Valk J., Brunner D., De Smet K., Svenningsen Å.F., Honegger P., Knudsen L.E., Lindl T., Noraberg J., Price A., Scarino M.L. (2010). Optimization of chemically defined cell culture media—Replacing fetal bovine serum in mammalian in vitro methods. Toxicol. Vitr..

[2] Yao T., Asayama Y. (2017). Animal-cell culture media: History, characteristics, and current issues. Reprod. Med. Biol..

[3] Jochems C.E.A., van der Valk J.B., Stafleu F.R., Baumans V. (2002). The Use of Fetal Bovine Serum: Ethical or Scientific Problem?. Altern. Lab. Anim..

[4] van der Valk J., Mellor D., Brands R., Fischer R., Gruber F., Gstraunthaler G., Hellebrekers L., Hyllner J., Jonker F., Prieto P. (2004). The humane collection of fetal bovine serum and possibilities for serum-free cell and tissue culture. Toxicol. Vitr..

[5] Gstraunthaler G. (2003). Alternatives to the use of fetal bovine serum: Serum-free cell culture. ALTEX.

[6] Bauman E., Granja P., Barrias C. (2018). Fetal bovine serum-free culture of endothelial progenitor cells-progress and challenges. J. Tissue Eng. Regen. Med..

[7] Burnouf T., Strunk D., Koh M.B., Schallmoser K. (2016). Human platelet lysate: Replacing fetal bovine serum as a gold standard for human cell propagation?. Biomaterials.

[8] Mirabet V., Solves P., Miñana M.D., Encabo A., Carbonell-Uberos F., Blanquer A., Roig R. (2007). Human platelet lysate enhances the proliferative activity of cultured human fibroblast-like cells from different tissues. Cell Tissue Bank..

[9] Hemeda H., Giebel B., Wagner W. (2014). Evaluation of human platelet lysate versus fetal bovine serum for culture of mesenchymal stromal cells. Cytotherapy.

[10] Johansson L., Klinth J., Holmqvist O., Ohlson S. (2003). Platelet lysate: A replacement for fetal bovine serum in animal cell culture?. Cytotechnology.

[11] Pons M., Nagel G., Zeyn Y., Beyer M., Laguna T., Brachetti T., Sellmer A., Mahboobi S., Conradi R., Butter F. (2018). Human platelet lysate as validated replacement for animal serum to assess chemosensitivity_suppl. ALTEX.

[12] Rauch C. (2011). Alternatives to the use of fetal bovine serum: Human platelet lysates as a serum substitute in cell culture media. ALTEX.

[13] Tylek T., Schilling T., Schlegelmilch K., Ries M., Rudert M., Jakob F., Groll J. (2019). Platelet lysate outperforms FCS and human serum for co-culture of primary human macrophages and hMSCs. Sci. Rep..

[14] Fazzina R., Iudicone P., Mariotti A., Fioravanti D., Procoli A., Cicchetti E., Scambia G., Bonanno G., Pierelli L. (2015). Culture of human cell lines by a pathogen-inactivated human platelet lysate. Cytotechnology.

[15] Ferruzza S. (2013). Serum-reduced and serum-free media for differentiation of Caco-2 cells. ALTEX.

[16] Sambuy Y., De Angelis I., Ranaldi G., Scarino M.L., Stammati A., Zucco F. (2005). The Caco-2 cell line as a model of the intestinal barrier: Influence of cell and culture-related factors on Caco-2 cell functional characteristics. Cell Biol. Toxicol..

[17] Van Breemen R.B., Li Y. (2005). Caco-2 cell permeability assays to measure drug absorption. Expert Opin. Drug Metab. Toxicol..

[18] Shah P., Jogani V., Bagchi T., Misra A. (2006). Role of Caco-2 Cell Monolayers in Prediction of Intestinal Drug Absorption. Biotechnol. Prog..

[19] Vandesompele J., De Preter K., Pattyn F., Poppe B., Van Roy N., De Paepe A., Speleman F. (2002). Accurate normalization of real-time quantitative RT-PCR data by geometric averaging of multiple internal control genes. Genome Biol..

[20] Schmitz J., Preiser H., Maestracci D., Ghosh B., Cerda J., Crane R. (1973). Purification of the human intestinal brush border membrane. Biochim. Biophys. Acta (BBA) Biomembr..

[21] Sterchi E.E., Woodley J.F. (1980). Peptide hydrolases of the human small intestinal mucosa: Identification of six distinct enzymes in the brush border membrane. Clin. Chim. Acta.

[22] Shimada Y., Inomata M., Suzuki H., Hayashi M., Waheed A.A., Ohno-Iwashita Y. (2005). Separation of a cholesterol-enriched microdomain involved in T-cell signal transduction. FEBS J..

[23] Naim H.Y., E Sterchi E., Lentze M.J. (1988). Biosynthesis of the human sucrase-isomaltase complex. Differential O-glycosylation of the sucrase subunit correlates with its position within the enzyme complex. J. Biol. Chem..

[24] Hauri H.P., Sterchi E.E., Bienz D., Fransen J., Marxer A. (1985). Expression and intracellular transport of microvillus membrane hydrolases in human intestinal epithelial cells. J. Cell Biol..

[25] Van Der Valk J. (2018). Fetal bovine serum (FBS): Past—Present—Future. ALTEX.

[26] Fernandez-Rebollo E., Mentrup B., Ebert R., Franzen J., Abagnale G., Sieben T., Ostrowska A., Hoffmann P., Roux P.-F., Rath B. (2017). Human Platelet Lysate versus Fetal Calf Serum: These Supplements do not Select for Different Mesenchymal Stromal Cells. Sci. Rep..

[27] Hemeda H., Kalz J., Walenda G., Lohmann M., Wagner W. (2013). Heparin concentration is critical for cell culture with human platelet lysate. Cytotherapy.

[28] Thieme D., Reuland L., Lindl T., Kruse F., Fuchsluger T. (2017). Optimized human platelet lysate as novel basis for a serum-, xeno-, and additive-free corneal endothelial cell and tissue culture. J. Tissue Eng. Regen. Med..

[29] Nepal N., Arthur S., Sundaram U. (2019). Unique Regulation of Na-K-ATPase during Growth and Maturation of Intestinal Epithelial Cells. Cells.

[30] Ranaldi G., Consalvo R., Sambuy Y., Scarino M.L. (2003). Permeability characteristics of parental and clonal human intestinal Caco-2 cell lines differentiated in serum-supplemented and serum-free media. Toxicol. Vitr..

[31] Zweibaum A., Triadou N., Kedinger M., Augeron C., Robine-Léon S., Pinto M., Rousset M., Haffen K. (1983). Sucrase-isomaltase: A marker of foetal and malignant epithelial cells of the human colon. Int. J. Cancer.

[32] Wetzel G., Heine M., Rohwedder A., Naim H.Y. (2009). Impact of glycosylation and detergent-resistant membranes on the function of intestinal sucrase-isomaltase. Biol. Chem..

[33] Toutounji M., Wanes D., El-Harakeh M., El-Sabban M., Rizk S., Naim H.Y. (2020). Dextran Sodium Sulfate-Induced Impairment of Protein Trafficking and Alterations in Membrane Composition in Intestinal Caco-2 Cell Line. Int. J. Mol. Sci..

[34] Alfalah M., Jacob R., Preuss U., Zimmer K.-P., Naim H., Naim H.Y. (1999). O-linked glycans mediate apical sorting of human intestinal sucrase-isomaltase through association with lipid rafts. Curr. Biol..

[35] Hashimoto K., Shimizu M. (1993). Epithelial properties of human intestinal Caco-2 cells cultured in a serum-free medium. Cytotechnology.

[36] Warrier A., Gunosewoyo H., Crowe A. (2018). Efflux transporters and tight junction expression changes in human gastrointestinal cell lines cultured in defined medium vs serum supplemented medium. Life Sci..

[37] Aldén A., Gonzalez L., Persson A., Christensson K., Holmqvist O., Ohlson S. (2007). Porcine platelet lysate as a supplement for animal cell culture. Cytotechnology.

[38] Hagen A., Lehmann H., Aurich S., Bauer N., Melzer M., Moellerberndt J., Patané V., Schnabel C.L., Burk J. (2021). Scalable Production of Equine Platelet Lysate for Multipotent Mesenchymal Stromal Cell Culture. Front. Bioeng. Biotechnol..

[39] Abu-Ameerh M.A., Jafar H., Hasan M.H., Al Bdour M., Msallam M., Ababneh O.H., Alhattab D.M., Al-Kurdi B., Awidi A., Awidi A.S. (2019). Platelet lysate promotes re-epithelialization of persistent epithelial defects: A pilot study. Int. Ophthalmol..

[40] Henschler R., Gabriel C., Schallmoser K., Burnouf T., Koh M.B. (2019). Human platelet lysate current standards and future developments. Transfusion.

